# Perforinopathy: A Spectrum of Human Immune Disease Caused by Defective Perforin Delivery or Function

**DOI:** 10.3389/fimmu.2013.00441

**Published:** 2013-12-12

**Authors:** Ilia Voskoboinik, Joseph A. Trapani

**Affiliations:** ^1^Killer Cell Biology Laboratory, Peter MacCallum Cancer Centre, East Melbourne, VIC, Australia; ^2^Sir Peter MacCallum Department of Oncology, The University of Melbourne, Melbourne, VIC, Australia; ^3^Cancer Cell Death Laboratory, Peter MacCallum Cancer Centre, East Melbourne, VIC, Australia

**Keywords:** perforin, perforinopathy, granzyme, NK cell, protein misfolding, FHL, immune deficiency

## Abstract

Congenital perforin deficiency is considered a rare cause of human immunopathology and immune dysregulation, and classically presents as a fatal illness early in infancy. However, we propose that a group of related disorders in which killer lymphocytes deliver only partially active perforin or a reduced quantum of wild-type perforin to the immune synapse should be considered part of an extended syndrome with overlapping but more variable clinical features. Apart from the many rare mutations scattered over the coding sequences, up to 10% of Caucasians carry the severely hypomorphic *PRF1* allele C272 > T (leading to A91V mutation) and the overall prevalence of the homozygous state for A91V is around 1 in 600 individuals. We therefore postulate that the partial loss of perforin function and its clinical consequences may be more common then currently suspected. An acute clinical presentation is infrequent in A91V heterozygous individuals, but we postulate that the partial loss of perforin function may potentially be manifested in childhood or early adulthood as “idiopathic” inflammatory disease, or through increased cancer susceptibility – either hematological malignancy or multiple, independent primary cancers. We suggest the new term “perforinopathy” to signify the common functional endpoints of all the known consequences of perforin deficiency and failure to deliver fully functional perforin.

## Introduction

Perforin (PRF, encoded by the *PRF1* gene) is a pore-forming toxin (1, 2) stored in the secretory granules of cytotoxic T lymphocytes (CTLs) and natural killer (NK) cells (collectively known as cytotoxic lymphocytes, CLs) (3). During an immune response, CLs form an immune synapse (IS) with various types of antigen-presenting cell, and release PRF and granzyme serine proteases into the synaptic cleft (4). The extracellular milieu of the IS (high calcium and neutral pH) promotes perforin membrane binding and insertion culminating in pore formation (5, 6), an indispensable requirement for granzymes to enter the target cell cytoplasm and trigger a multitude of cell death signaling pathways (7–12).

## A Spectrum of Clinical Presentations Reflecting Functional PRF Deficiency

It was first appreciated around the turn of this century that the complete loss of perforin activity results in a fatal, autosomal recessive immunoregulatory disorder of infancy (median age of onset <12 months), familial hemophagocytic lymphohistiocytosis (FHL) (13), which can be cured only by heterologous bone marrow transplantation (14). The inability to clear antigen-presenting cells by impaired CLs causes an uncontrolled activation and expansion of CD4+ and CD8+ T cells that secrete high levels of interferon-γ. This cytokine is central to disease pathogenesis, as it leads to macrophage activation and secondarily to the overproduction of pro-inflammatory cytokines by these cells. This in turn is manifested clinically as intractable fever, liver and spleen enlargement, and hemophagocytosis in the bone marrow and lymphoid organs, leading to severe anemia and leukopenia (15). It is generally thought that macrophage activation and infiltration in bone marrow leads to hemophagocytosis in FHL patients. Recently, it was shown that interferon-γ specifically potentiated phagocytosis of erythrocytes by macrophages, thus suggesting a key mechanism for anemia in these patients (16). Variations in clinical presentation stem in part from the fact that there are up to five independent genetic causes of primary FHL and related syndromes (4). Linkage analyses have identified four genetic causes that account for 80–90% of FHL, while a further locus mapping to Chr 9 is yet to be defined (17). Inactivating perforin mutations are responsible for approximately 50% of all cases (type 2 FHL, FHL2), while mutations in three other genes, *UNC13D, STX11*, and *STXBP2* (FHL3, 4, and 5 respectively) impair or ablate the delivery of perforin to the IS (18–21). Thus, all the causes of FHL are causally linked by the failure to deliver sufficient active perforin to the IS.

Natural killer cells (innate immunity) and CTLs (adaptive immunity) were first recognized for their key role/s in the defense against viruses; more recently these cells have also been appreciated as being critical for immune surveillance against a variety of malignancies, particularly those of hematopoietic origin (22, 23) or of multiple cancers in the same individuals (24). Both these roles reflect the importance of perforin in initiating the apoptosis of dangerous cells, either those harboring an intracellular pathogen, or possessing the potential for uninhibited growth and spread, to the detriment of the host. Given this, it is perhaps surprising that (infantile) FHL rarely presents with overwhelming sepsis syndrome caused by viral infection (with the rare exceptions of persistent or fulminant Epstein–Barr virus infection) and almost never with malignancy. Thus, most cases of FHL are not manifested directly through the consequences of failed “target cell death” but rather, indirectly, through an apparent skewing of the immune response toward exaggerated cytokine secretion, the second major type of effector function of CTL/NK cells.

Recent work on CTL/NK cells has highlighted their regulation of various inflammatory pathways through their cross-talk with other components of the immune system (25–27); the consequent aberrations in inflammatory responses leading from failed perforin production can occur in response to known human pathogens, although a specific pathogen that triggers the onset of FHL is rarely identified. These phenomena strongly indicate that perforin and the pathways that synthesize and deliver it to the IS play a more fundamental role in immune homeostasis, centered more particularly on adaption of the neonate to the myriad “non-pathogenic” antigenic stimuli he/she will encounter after leaving the womb. In this context, we postulate it is the failure to clear “constitutive” antigen-presenting cells through perforin-dependent (largely granzyme-mediated) cell death that induces the increased secretion of interferon-γ from the killer lymphocyte, in turn provoking severe dysregulation of pro-inflammatory and chemokine cascades in by-stander cells. The molecular and cellular mechanisms causally linking failed target cell apoptosis and the hypersecretion of interferon-γ are of fundamental importance to the pathophysiology of perforinopathies, and ought to be the focus of intensive research. The consequential effects on macrophages, including secondary pro-inflammatory cytokine secretion and grossly increased macrophage phagocytic activity are the major manifestations of disease and are most marked when perforin activity is completely abolished, but less so if some residual perforin activity persists, for example due to inheritance of perforin missense mutation/s that are not completely inactivating. This theme is taken up again below, as we believe that the variable clinical presentations probably flow on from this fact. While this paper focuses on perforin, it has also been noted for some time that granzymes (for example, granzymes A and M) can influence inflammatory pathways (25, 27), sometimes in a perforin-dependent manner, and at other times without the need for perforin to be present. While the significance of granzyme-mediated inflammatory pathways for human health are yet to be defined and hyper- (or hypo-) inflammatory syndromes are not yet described, future research should keep this possibility in mind. This is particularly so because granzyme genes can show considerable polymorphism, both in mice (28) and humans (29).

**Figure 1:**
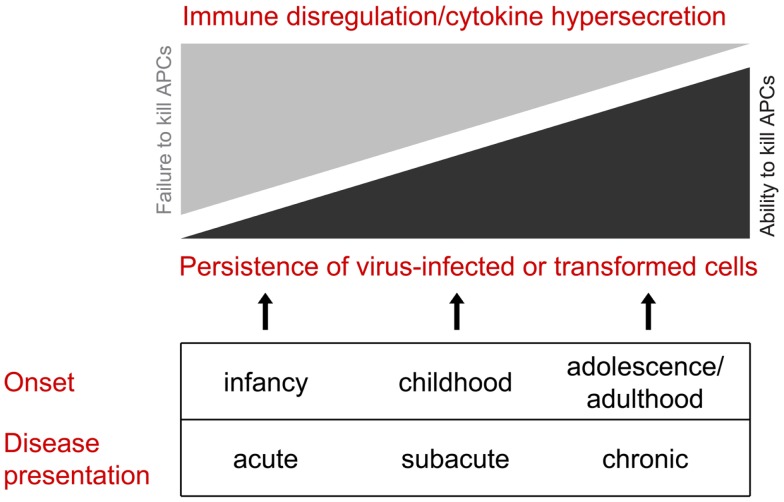
**All of the clinical presentations of perforin deficiency (including the failure to deliver active perforin into the immune synapse) result from failed killing of an antigen-presenting cell**. Early in life, the clinical manifestations are more likely to reflect cytokine hypersecretion due to chronic stimulation of CD4 and CD8 CTLs and NK cells, resulting in disordered immune hemostasis affected both the lymphoid and myeloid compartments. Later in life, hyper-inflammatory presentations are less likely, and the clinical scenario is more likely to reflect failure to clear dangerous target cells, particularly virus-infected cells and neoplastic cells.

On the basis that congenital defects in CTL/NK that influence the secretion of active perforin may become clinically evident at various stages of life, we would like to propose that this group of disorders be considered as perforinopathies under three subheadings: acute, subacute, and chronic, depending on the stage of disease onset (Table 1). As alluded to above, earlier presentations tend to reflect cytokine-mediated immunopathology; later in life, a patient may present more cryptically with relatively weaker inflammatory manifestations or none at all (Figure 1). We think it is quite likely that clinicians managing these less urgent clinical presentations may not have previously considered perforinopathy in the differential diagnosis, particularly in children presenting with a variety of inflammatory disorders beyond infancy.

**Table 1:** **Possible clinical manifestations of perforinopathy**.

	Acute perforinopathy	Sub-acute perforinopathy	Chronic perforinopathy
Age at onset	0–2 years	>2 years	Adolescents – adults
Cause	Bi-allelic mutations in *PRF1, UNC13D, STX11, STXBP2*, leading to complete loss-of-function	Bi-allelic mutations in *PRF1* and, putatively, in *UNC13D, STX11, STXBP2*, leading to partial loss-of-function	Monoallelic mutations in *PRF1* [e.g., polymorphism 272C > T (Ala91Val)], and possibly, other FHL-causing genes, are contributing not causative factors
Onset	“Classical” FHL – meets all or most of the criteria described in HLH-2004	Difficult to diagnose as does not meet minimal essential criteria of HLH-2004: e.g., inflammatory disease that responds to corticosteroid therapy and has remitting/relapsing clinical course; hematological malignancy	May include a range of conditions, including hematological malignancy, macrophage activation syndrome, lymphoproliferative disease, but not FHL
Diagnostic features	Intractable fever and hepatosplenomegaly are early and prominent	Once FHL is suspected, conduct tests as described in HLH-2004. Severe reduction of NK cell function that prompts genetic analysis may indicate FHL. Test siblings for bi-allelic mutations in FHL-related genes	Mild reduction of NK function. No symptoms described in HLH-2004 are expected
	Investigations (including NK function assays) described in HLH-2004 will strongly suggest FHL, and disease is confirmed by DNA sequencing	
Therapy	Use protocols in HLH-2004; heterologous stem cell transplantation is the only curative therapy	Initially, corticosteroid therapy	Disease-specific therapy, ranging from corticosteroids to stem cell transplantation
		When genetic cause is identified, HLH-2004 and heterologous stem cell transplantation, as the only cure. If asymptomatic siblings are carriers of bi-allelic mutations in FHL-related genes, preventative stem cell transplantation may be considered	

## *Acute* Presentations of Perforinopathy – FHL

For the purpose of this discussion, we define acute perforinopathy as that resulting in a clinical presentation prior to the age of 24 months, with median of approximately 9 months (this may vary depending on the genetic cause of FHL). As discussed above, the pathogenesis involves a cascade of downstream events following on from the inability of NK cells and CTL to present functional perforin and, therefore, kill a cognate target cell. Affected infants typically present with “classic” FHL and meet all or most of the criteria described in HLH-2004 (15). The diagnosis is confirmed by the loss of NK cytotoxicity (which is convenient to test, as the NK cells of healthy individuals display constitutive, pathogen-independent cytotoxicity) and identification of mutations in candidate genes (*PRF1, UNC13D, STX11, STXBP2*). Affected infants are typically very unwell, and may require admission to a high-dependency or intensive care unit; given the autosomal recessive inheritance of most of these disorders, a sibling may have previously been similarly affected, while parents are almost always unaffected carriers. Patients first have their condition stabilized, and are then prepared for heterologous bone marrow transplantation, the only potentially curative therapy (14, 15).

Acute perforinopathy is caused by detrimental mutations in *PRF1* or in proteins responsible for its delivery from the lumen of cytotoxic granules to the IS, *UNC13D, STX11*, or *STXBP2* (4). The molecular basis of many disease-causing *PRF1* mutations has been investigated directly using recombinant expression systems (23, 30–33), and is supported by the X-ray crystal structure of perforin (1). In contrast, the biochemical bases of pathological mutations in the other three proteins, Munc13-4, Syntaxin11, and Munc18-2, remain largely unexplored due to the lack of sufficiently informative experimental systems. As a result, the loss of molecular function is predicted (34, 35), either on the basis of undetectable NK cell activity and/or severely reduced protein expression due to degradation. In the context of an early onset FHL (acute perforinopathy), the known structural and cellular defects correlate well with disease severity. Indeed, in the case of *PRF1*, genuinely “null” mutations are most commonly caused by nonsense or frame-shift mutations and in-frame deletions that completely abrogate function. By contrast, a careful genotype-phenotype analysis has revealed that only about 50% of missense mutations ever reported resulted in a complete loss of perforin function, either due to direct interference with a critical functional domain or unscheduled post-translational modifications such as glycosylation (30, 36). The remaining *PRF1* missense mutations were more commonly associated with atypical presentations of FHL or with seemingly unrelated pathologies (immunoregulatory or otherwise) in older children, adolescents, and even adults, who did not necessarily presented with FHL at all (23, 37). If residual perforin activity delays the clinical onset beyond the age of 12 months, we term the presentation “subacute.”

## *Sub-acute* Presentations of Perforinopathy

Sub-acute perforinopathies have a wide spectrum of manifestations, all of which are all caused by a partial (“sub-total”) loss of CL cytotoxicity due to bi-allelic mutations in one of the four genes described above. Unlike the acute form of the disease, sub-acute perforinopathies may be difficult to diagnose due to their generally milder and more “patchy” clinical presentations, an intermittent clinical course, a range of ages of onset and their frequent response to non-specific immune-suppressive or immune-ablative therapies (38, 39). These patients are more likely to present with a precipitating infection or with an isolated inflammatory manifestation such as pneumonitis that can lead the treating clinician away from the underlying diagnosis. While the only curative therapy for such patients is still ultimately bone marrow transplantation, combination drug therapy may induce remissions of variable duration. These non-curative interventions may be the only available therapies that influence disease outcome, but they have frequent unwanted effects such as opportunistic infection, growth retardation, and bone marrow suppression. Alleviating symptoms that might have categorized them as FHL patients might also delay diagnosis in both the patient and their younger siblings (38). Considering the fact that “classic” FHL is relatively rare and commonly described in infants or very young children, we believe sub-acute perforinopathies may be under-diagnosed, and thus may delay consideration of curative stem cell transfer as a definitive treatment option.

How common are sub-acute perforinopathies? The inevitable answer is “We do not know.” Due to the lack of direct functional data on Munc13-4, syntaxin 11, and Munc18-2 mutations, we will discuss perforin deficiency as a paradigm for partial loss of CL function. The first indication that bi-allelic perforin mutations may lead to atypical FHL came from the observation that individuals who inherit two mutant alleles of *PRF1*, one of which is the common polymorphism C272 > T, encoding the ostensibly conservative Ala91Val (A91V) substitution, presented at an older age than “classic” FHL, and with variable symptoms (13, 38, 39). The initial suggestion that the “polymorphism” may be pathogenic was initially met with skepticism, due largely to the very high frequency of the allele among Caucasians (8–17% are heterozygotes) and the rareness of early onset FHL, which was considered at that time to be the only authentic manifestation of the disease (40). However, over the next several years, additional clinical research and biochemical studies have confirmed the original prediction that the A91V substitution is a genuine mutation that impacts severely on perforin structure and function (31, 41). While a prospective cohort study has never been reported, it is nonetheless clear from many reported case studies that inheritance of A91V in the homozygous state or its co-inheritance with a genuinely null allele is very strongly associated with atypical FHL or other immunopathologies that are delayed compared with “classic” disease (sub-acute perforinopathies). In the largest epidemiological studies reported to date [involving over 2,600 healthy individuals – (42–44)], healthy A91V homozygotes have been reported only in one study (42); every other reported case of A91V homozygocity was invariably associated with a pathology. However, the statistical power of these studies linking the genotype with disease has been insufficient to achieve significance. Based on the Mendelian inheritance of perforin mutations, it is predicted that at least 1 in 600 Caucasian individuals should be homozygous for A91V. Assuming strong penetrance of an associated immunopathology in these individuals, such a perforinopathy would be almost 100 times as common as classic (“acute”) FHL. Remarkably, at this frequency, A91V-related immune deficiency would rank among the most common congenital disorders; it would be approximately four times as frequent as cystic fibrosis and at a par with Down syndrome.

Is partial perforin deficiency a common phenomenon, and is it caused only by A91V? A retrospective analysis of all reported cases of FHL associated with bi-allelic *PRF1* mutations uncovered a previously unappreciated dichotomy: half of the patients who carried at least one missense mutation in *PRF1* presented “acutely,” with a FHL-like syndrome before the age of 12 months, while the remaining 50% had significantly delayed FHL, or presented with other, seemingly unrelated pathologies (23, 37, 45). Most of these patients either developed hematological malignancies (usually beyond the age of 10 years, which we consider to be a “chronic” presentation) or presented with unusual or protracted viral infections (23, 46, 47). Critically, biochemical analysis of multiple missense mutations associated with atypical FHL has led to the realization that partial perforin deficiency results from perforin misfolding: all reported cases of atypical, delayed FHL had some recoverable perforin-mediated cytotoxicity *in vitro* (23). Structural studies recently supported these findings, as wild-type perforin was shown to be a thermo-labile protein, with a melting temperature (the temperature at which the protein denatures and starts to lose function) only slightly above 40°C (5). Even though this analysis was conducted *in vitro*, this finding has raised the intriguing possibility that the protracted, cytokine-induced fever of FHL might further aggravate the disease by accentuating the protein misfolding defect. Given the diversity of clinical presentation that encompasses cancer, atypical onset of viral infections, and attenuated FHL-like syndromes, it is likely that a significant proportion of sub-acute perforinopathies go undiagnosed.

Identification of missense mutations that confer partial activity for the proteins encoded by the *UNC13D, STX11*, and *STXBP2* genes may broaden the spectrum of recognized sub-acute perforinopathies. A number of non-synonymous polymorphisms in these genes have already been cataloged in single nucleotide polymorphism (SNP) databases, and future studies will determine whether any of these variations can affect the rate and/or efficiency of secretory granule exocytosis and membrane fusion, steps that ultimately determine the amount of perforin secreted into the IS and its rate of delivery.

## *Chronic* Presentations of Perforinopathy

We consider chronic perforinopathies to be disease states that represent as a spectrum of immune-mediated diseases associated with monoallelic mutations in FHL-related genes. The presentations typically do not have a strong resemblance to classic FHL and may include blood cancers and macrophage activation syndrome in patients with juvenile rheumatoid arthritis. Typically, age of onset will be beyond 5 years of age. In addition, some studies have reported an association of *PRF1* polymorphisms and the outcome of allogeneic bone marrow transplantation. All of these associations are contentious, as studies that find no link outnumber those supporting such an association.

Once again, the most reliable and “testable” information comes from the analysis of *PRF1* polymorphisms, as the functional consequence of mutations is well understood. Considering the fact that hundreds of FHL patients with known genetic defects have been diagnosed around the world since 1999, the number of their family members who are known carriers of monoallelic mutations, should exceed 1,000. This is a significant cohort of unrelated individuals, who may be potentially investigated in prospective longitudinal studies on immune surveillance of infections and cancer and for dysregulated immune homeostasis.

An association between presumed partial CL deficiency due to monoallelic A91V perforin polymorphism, and acute lymphoblastic leukemia or macrophage activation syndrome, has been demonstrated in several studies that have necessarily involved a limited number of individuals (48, 49), but no statistical difference was observed in follow up studies when larger cohorts of patients were examined (24, 43). The likely reason for this apparent discrepancy may be the relatively low statistical power of smaller studies. Another possibility is that a partial deficiency of CL function may predispose more strongly to a specific subtype of malignancy or other pathology. For example, while the largest analysis of patents with acute lymphoblastic leukemia did not reveal an enrichment of A91V carriers compared to healthy controls, a subset of the ALL patients who also had BCR-ABL translocations were more likely to carry the A91V allele than the control group (43). Similarly, in a study that retrospectively examined patients who had been diagnosed with more than one primary malignancy during their lifetime, a significant proportion of patients diagnosed with both melanoma and B cell lymphoma were carriers of A91V or another perforin mutation that is partly inactivating (R28C), than those who had been diagnosed with either disease alone (24).

We have tried to explore the molecular basis for how a monoallelic mutation might adversely affect overall cytotoxic function in an inherited condition classically considered to be recessive. Molecular analysis has revealed that A91V has a partial dominant-negative effect on the function of wild-type perforin; partly misfolded perforin might interfere with the expression or trafficking of normal perforin through the endoplasmic reticulum/Golgi to the secretory granules (31, 50). Once wild-type and misfolded perforin molecules are “mixed” and stored together, it is easy to imagine that perforin pores with a heterogeneous composition of monomers might have disproportionately poor function. We also speculate that in heterozygous individuals, the wild-type allele may be under-expressed relative to the mutated allele, thus further diminishing overall CL function. This possibility needs to be formally excluded, but has been described for many other polymorphic genes [e.g., Ref. (51)].

Further analysis of CL function in patients with immune-mediated disease is warranted, particularly employing recent advances in single-cell microscopy technology, which may reveal CL deficiencies that would otherwise remain undetected in cell population-based experiments (6). Such technologies may validate the more common statistical approaches, where for example, the frequency of a *PRF1* mutation in cancer patients is compared to that in healthy controls. In acute perforinopathy, there is a clear causal relationship between the disease phenotype and the patient’s genotype, but this is not the case in cancer (even assuming a clinician considered testing for perforin genotype, which would be unusual). Rather, most chronic perforinopathies are likely to present “indirectly.” In cancer, the *PRF1* gene [and, most likely, *UNC13D, STX11*, and *STXBP2* (52)] would be considered to be a tumor suppressor gene, but is not the cause of malignancy *per se* and is certainly not the only causal factor. Rather, impaired perforin function reduces the surveillance of cancerous cells, which were generated through an unrelated genetic event. This notion raises a question of what constitutes an appropriate control in these studies. Since the hypothesis is that carriers of monoallelic mutation(s) in *PRF1* or related genes predispose to immune-mediated diseases with uncertain age of onset, taking a “snapshot” of a healthy population may underestimate the impact of mutations. In contrast, it was recently shown that individual differences between the cytotoxic activity of NK cells (and cytotoxic T cells) are much smaller than previously thought (6). This opens an avenue for “personalized” analysis of CL function that may shed light on predisposition of an individual to an immune pathology, prospectively and retrospectively.

## Summary

In this discourse, we have proposed the term “perforinopathy” to denote the wide and diverse spectrum of manifestations of perforin deficiency, both temporal and clinical. Early (acute) clinical presentations are often fatal and usually represent dysregulated immune homeostasis and the resultant hypercytokinemia (“cytokine storm”) and hemophagocytosis consequent on macrophage activation. Sub-acute presentations still occur in infancy/early childhood, may typically have a more benign course that is still principally inflammatory and may accompany other pathologies such as juvenile idiopathic arthritis or virus infection. Chronic manifestations of perforin deficiency may appear as late as adulthood, and the principal cause of symptoms is the failure to clear specific dangerous cells, particularly pre-malignant cells. Hematopoietic malignancies seem to be a common outcome, and some patients may present well into adulthood with more than one primary cancer: early onset malignant melanoma and lymphoma is the commonest combination we have observed. Although most of the data supporting our proposed “classification” is centered on our knowledge of *PRF1* gene mutations, we hope that this paper will stimulate further study on polymorphisms and mutations of other genes that play a role in delivering functional perforin to the target cell.

## Conflict of Interest Statement

The authors declare that the research was conducted in the absence of any commercial or financial relationships that could be construed as a potential conflict of interest.

## References

[1] Law RH Lukoyanova N Voskoboinik I Caradoc-Davies TT Baran K Dunstone MA The structural basis for membrane binding and pore formation by lymphocyte perforin. Nature (2010) 468:447–51 10.1038/nature09518 21037563

[2] Baran K Dunstone M Chia J Ciccone A Browne KA Clarke CJ The molecular basis for perforin oligomerization and transmembrane pore assembly. Immunity (2009) 30:684–95 10.1016/j.immuni.2009.03.016 19446473

[3] Peters PJ Borst J Oorschot V Fukuda M Krahenbuhl O Tschopp J Cytotoxic T lymphocyte granules are secretory lysosomes, containing both perforin and granzymes. J Exp Med (1991) 173:1099–109 10.1084/jem.173.5.1099 2022921 PMC2118839

[4] de Saint Basile G Gaël Ménasché G Fischer A Molecular mechanisms of biogenesis and exocytosis of cytotoxic granules. Nat Rev Immunol (2010) 11:568–79 10.1038/nri2803 20634814

[5] Lopez JA Susanto O Jenkins MR Lukoyanova N Sutton VR Law RH Perforin forms transient pores on the target cell plasma membrane to facilitate rapid access of granzymes during killer cell attack. Blood (2013) 121:2659–68 10.1182/blood-2012-07-446146 23377437

[6] Lopez JA Jenkins MR Rudd-Schmidt JA Brennan AJ Danne JC Mannering SI Rapid and unidirectional perforin pore delivery at the cytotoxic immune synapse. J Immunol (2013) 191:2328–34 10.4049/jimmunol.1301205 23885110

[7] Walsh CM Matloubian M Liu CC Ueda R Kurahara CG Christensen JL Immune function in mice lacking the perforin gene. Proc Natl Acad Sci U S A (1994) 91:10854–8 10.1073/pnas.91.23.10854 7526382 PMC45124

[8] Su B Bochan MR Hanna WL Froelich CJ Brahmi Z Human granzyme B is essential for DNA fragmentation of susceptible target cells. Eur J Immunol (1994) 24:2073–80 10.1002/eji.1830240921 8088328

[9] Shiver JW Su L Henkart PA Cytotoxicity with target DNA breakdown by rat basophilic leukemia cells expressing both cytolysin and granzyme A. Cell (1992) 71:315–22 10.1016/0092-8674(92)90359-K 1423596

[10] Shi L Kraut RP Aebersold R Greenberg AH A natural killer cell granule protein that induces DNA fragmentation and apoptosis. J Exp Med (1992) 175:553–66 10.1084/jem.175.2.553 1732416 PMC2119135

[11] Shi L Kam CM Powers JC Aebersold R Greenberg AH Purification of three cytotoxic lymphocyte granule serine proteases that induce apoptosis through distinct substrate and target cell interactions. J Exp Med (1992) 176:1521–9 10.1084/jem.176.6.1521 1460416 PMC2119451

[12] Heusel JW Wesselschmidt RL Shresta S Russell JH Ley TJ Cytotoxic lymphocytes require granzyme B for the rapid induction of DNA fragmentation and apoptosis in allogeneic target cells. Cell (1994) 76:977–87 10.1016/0092-8674(94)90376-X 8137431

[13] Stepp SE Dufourcq-Lagelouse R Le Deist F Bhawan S Certain S Mathew PA Perforin gene defects in familial hemophagocytic lymphohistiocytosis. Science (1999) 286:1957–9 10.1126/science.286.5446.1957 10583959

[14] Janka GE Familial and acquired hemophagocytic lymphohistiocytosis. Annu Rev Med (2012) 63:233–46 10.1146/annurev-med-041610-134208 22248322

[15] Henter JI Horne A Arico M Egeler RM Filipovich AH Imashuku S HLH-2004: diagnostic and therapeutic guidelines for hemophagocytic lymphohistiocytosis. Pediatr Blood Cancer (2007) 48:124–31 10.1002/pbc.21039 16937360

[16] Zoller EE Lykens JE Terrell CE Aliberti J Filipovich AH Henson PM Hemophagocytosis causes a consumptive anemia of inflammation. J Exp Med (2011) 208:1203–14 10.1084/jem.20102538 21624938 PMC3173248

[17] Ohadi M Lalloz MR Sham P Zhao J Dearlove AM Shiach C Localization of a gene for familial hemophagocytic lymphohistiocytosis at chromosome 9q21.3-22 by homozygosity mapping. Am J Hum Genet (1999) 64:165–71 10.1086/302187 9915955 PMC1377714

[18] zur Stadt U Schmidt S Kasper B Beutel K Diler AS Henter JI Linkage of familial hemophagocytic lymphohistiocytosis (FHL) type-4 to chromosome 6q24 and identification of mutations in syntaxin 11. Hum Mol Genet (2005) 14:827–34 10.1093/hmg/ddi076 15703195

[19] zur Stadt U Rohr J Seifert W Koch F Grieve S Pagel J Familial hemophagocytic lymphohistiocytosis type 5 (FHL-5) is caused by mutations in Munc18-2 and impaired binding to syntaxin 11. Am J Hum Genet (2009) 85:482–92 10.1016/j.ajhg.2009.09.005 19804848 PMC2756548

[20] Feldmann J Callebaut I Raposo G Certain S Bacq D Dumont C Munc13-4 is essential for cytolytic granules fusion and is mutated in a form of familial hemophagocytic lymphohistiocytosis (FHL3). Cell (2003) 115:461–73 10.1016/S0092-8674(03)00855-9 14622600

[21] Cote M Menager MM Burgess A Mahlaoui N Picard C Schaffner C Munc18-2 deficiency causes familial hemophagocytic lymphohistiocytosis type 5 and impairs cytotoxic granule exocytosis in patient NK cells. J Clin Invest (2009) 119:3765–73 10.1172/JCI40732 19884660 PMC2786810

[22] Clementi R Locatelli F Dupre L Garaventa A Emmi L Bregni M A proportion of patients with lymphoma may harbor mutations of the perforin gene. Blood (2005) 105:4424–8 10.1182/blood-2004-04-1477 15728124

[23] Chia J Yeo KP Whisstock JC Dunstone MA Trapani JA Voskoboinik I Temperature sensitivity of human perforin mutants unmasks subtotal loss of cytotoxicity, delayed FHL, and a predisposition to cancer. Proc Natl Acad Sci U S A (2009) 106:9809–14 10.1073/pnas.0903815106 19487666 PMC2701033

[24] Trapani JA Thia KYT Andrews M Davis ID Gedye C Parente P Human perforin mutations and susceptibility to multiple primary cancers. Oncoimmunology (2013) 2:e24185 10.4161/onci.24185 23734337 PMC3654607

[25] Anthony DA Andrews DM Chow M Watt SV House C Akira S A role for granzyme M in TLR4-driven inflammation and endotoxicosis. J Immunol (2010) 185:1794–803 10.4049/jimmunol.1000430 20585036

[26] Anthony DA Andrews DM Watt SV Trapani JA Smyth MJ Functional dissection of the granzyme family: cell death and inflammation. Immunol Rev (2010) 235:73–92 10.1111/j.0105-2896.2010.00907.x 20536556

[27] Metkar SS Menaa C Pardo J Wang B Wallich R Freudenberg M Human and mouse granzyme A induce a proinflammatory cytokine response. Immunity (2008) 29:720–33 10.1016/j.immuni.2008.08.014 18951048

[28] Thia KY Trapani JA The granzyme B gene is highly polymorphic in wild mice but essentially invariant in common inbred laboratory strains. Tissue Antigens (2007) 70:198–204 10.1111/j.1399-0039.2007.00872.x 17661907

[29] McIlroy D Cartron PF Tuffery P Dudoit Y Samri A Autran B A triple-mutated allele of granzyme B incapable of inducing apoptosis. Proc Natl Acad Sci U S A (2003) 100:2562–7 10.1073/pnas.0437935100 12594335 PMC151380

[30] Chia J Thia K Brennan AJ Little M Williams B Lopez JA Fatal immune dysregulation due to a gain of glycosylation mutation in lymphocyte perforin. Blood (2012) 119:1713–6 10.1182/blood-2011-08-374355 22186995

[31] Voskoboinik I Sutton VR Ciccone A House CM Chia J Darcy PK Perforin activity and immune homeostasis: the common A91V polymorphism in perforin results in both presynaptic and postsynaptic defects in function. Blood (2007) 110:1184–90 10.1182/blood-2007-02-072850 17475905

[32] Voskoboinik I Thia M-C De Bono A Browne K Cretney E Jackson JT The functional basis for hemophagocytic lymphohistiocytosis in a patient with co-inherited missense mutations in the perforin (*PFN1*) gene. J Exp Med (2004) 200:811–6 10.1084/jem.20040776 15365097 PMC2211966

[33] Voskoboinik I Thia MC Trapani JA A functional analysis of the putative polymorphisms A91V and N252S and 22 missense perforin mutations associated with familial hemophagocytic lymphohistiocytosis. Blood (2005) 105:4700–6 10.1182/blood-2004-12-4935 15755897

[34] Sieni E Cetica V Santoro A Beutel K Mastrodicasa E Meeths M Genotype-phenotype study of familial haemophagocytic lymphohistiocytosis type 3. J Med Genet (2011) 48:343–52 10.1136/jmg.2010.085456 21248318 PMC4115201

[35] Pagel J Beutel K Lehmberg K Koch F Maul-Pavicic A Rohlfs AK Distinct mutations in STXBP2 are associated with variable clinical presentations in patients with familial hemophagocytic lymphohistiocytosis type 5 (FHL5). Blood (2012) 119:6016–24 10.1182/blood-2011-12-398958 22451424

[36] Al-Jasmi F Abdelhaleem M Stockley T Lee K-S Clarke JTR Novel mutation of the perforin gene and maternal uniparental disomy 10 in a patient with familial hemophagocytic lymphohistiocytosis. J Pediatr Hematol Oncol (2008) 30:621–4 10.1097/MPH.0b013e31817580fd 18799942

[37] Voskoboinik I Smyth MJ Trapani JA Perforin-mediated target-cell death and immune homeostasis. Nat Rev Immunol (2006) 6:940–52 10.1038/nri1983 17124515

[38] Busiello R Adriani M Locatelli F Galgani M Fimiani G Clementi R Atypical features of familial hemophagocytic lymphohistiocytosis. Blood (2004) 103:4610–2 10.1182/blood-2003-10-3551 14739222

[39] Clementi R Emmi L Maccario R Liotta F Moretta L Danesino C Adult onset and atypical presentation of hemophagocytic lymphohistiocytosis in siblings carrying PRF1 mutations. Blood (2002) 100:2266–7 10.1182/blood-2002-04-1030 12229880

[40] Zur Stadt U Beutel K Weber B Kabisch H Schneppenheim R Janka G A91V is a polymorphism in the perforin gene not causative of an FHLH phenotype. Blood (2004) 104:1909–10 10.1182/blood-2004-02-0733 15342365

[41] Trambas C Gallo F Pende D Marcenaro S Moretta L De Fusco C A single amino acid change, A91V, leads to conformational changes that can impair processing to the active form of perforin. Blood (2005) 106:932–7 10.1182/blood-2004-09-3713 15741215

[42] Orilieri E Cappellano G Clementi R Cometa A Ferretti M Cerutti E Variations of the perforin gene in patients with type 1 diabetes. Diabetes (2008) 57:1078–83 10.2337/db07-0947 18198357

[43] Mehta PA Davies SM Kumar A Devidas M Lee S Zamzow T Perforin polymorphism A91V and susceptibility to B-precursor childhood acute lymphoblastic leukemia: a report from the Children’s Oncology Group. Leukemia (2006) 20:1539–41 10.1038/sj.leu.2404299 16791263 PMC2922049

[44] Cappellano G Orilieri E Comi C Chiocchetti A Bocca S Boggio E Variations of the perforin gene in patients with multiple sclerosis. Genes Immun (2008) 9:438–44 10.1038/gene.2008.35 18496551

[45] Voskoboinik I Dunstone MA Baran K Whisstock JC Trapani JA Perforin: structure, function, and role in human immunopathology. Immunol Rev (2010) 235:35–54 10.1111/j.0105-2896.2010.00896.X 20536554

[46] Beaty AD Weller C Levy B Vogler C Ferguson WS Bicknese A A teenage boy with late onset hemophagocytic lymphohistiocytosis with predominant neurologic disease and perforin deficiency. Pediatr Blood Cancer (2007) 50:1070–2 10.1002/pbc.21438 18074390

[47] Katano H Ali MA Patera AC Catalfamo M Jaffe ES Kimura H Chronic active Epstein-Barr virus infection associated with mutations in perforin that impair its maturation. Blood (2004) 103:1244–52 10.1182/blood-2003-06-2171 14576041

[48] Cannella S Santoro A Bruno G Pillon M Mussolin L Mangili G Germline mutations of the perforin gene are a frequent occurrence in childhood anaplastic large cell lymphoma. Cancer (2007) 109:2566–71 10.1002/cncr.22718 17477373

[49] Santoro A Cannella S Trizzino A Lo Nigro L Corsello G Arico M A single amino acid change A91V in perforin: a novel, frequent predisposing factor to childhood acute lymphoblastic leukemia? Haematologica (2005) 90:697–8 15921391

[50] Brennan AJ Chia J Browne KA Ciccone A Ellis S Lopez JA Protection from endogenous perforin: glycans and the C terminus regulate exocytic trafficking in cytotoxic lymphocytes. Immunity (2011) 34:879–92 10.1016/j.immuni.2011.04.007 21658975

[51] Gartner JJ Parker SC Prickett TD Dutton-Regester K Stitzel ML Lin JC Whole-genome sequencing identifies a recurrent functional synonymous mutation in melanoma. Proc Natl Acad Sci U S A (2013) 110:13481–6 10.1073/pnas.1304227110 23901115 PMC3746936

[52] Machaczka M Klimkowska M Chiang SC Meeths M Muller ML Gustafsson B Development of classical Hodgkin’s lymphoma in an adult with biallelic STXBP2 mutations. Haematologica (2013) 98:760–4 10.3324/haematol.2012.073098 23100279 PMC3640121

